# Supplementary figures and images for: Non-linear genetic diversity and notable population differentiation caused by low gene flow of bermudagrass [Cynodon dactylon (L.) Pers.] along longitude gradients
Source: PeerJ. 2021 Aug 17;9:e11953. doi: 10.7717/peerj.11953 (PMC8378333; doi:10.7717/peerj.11953)

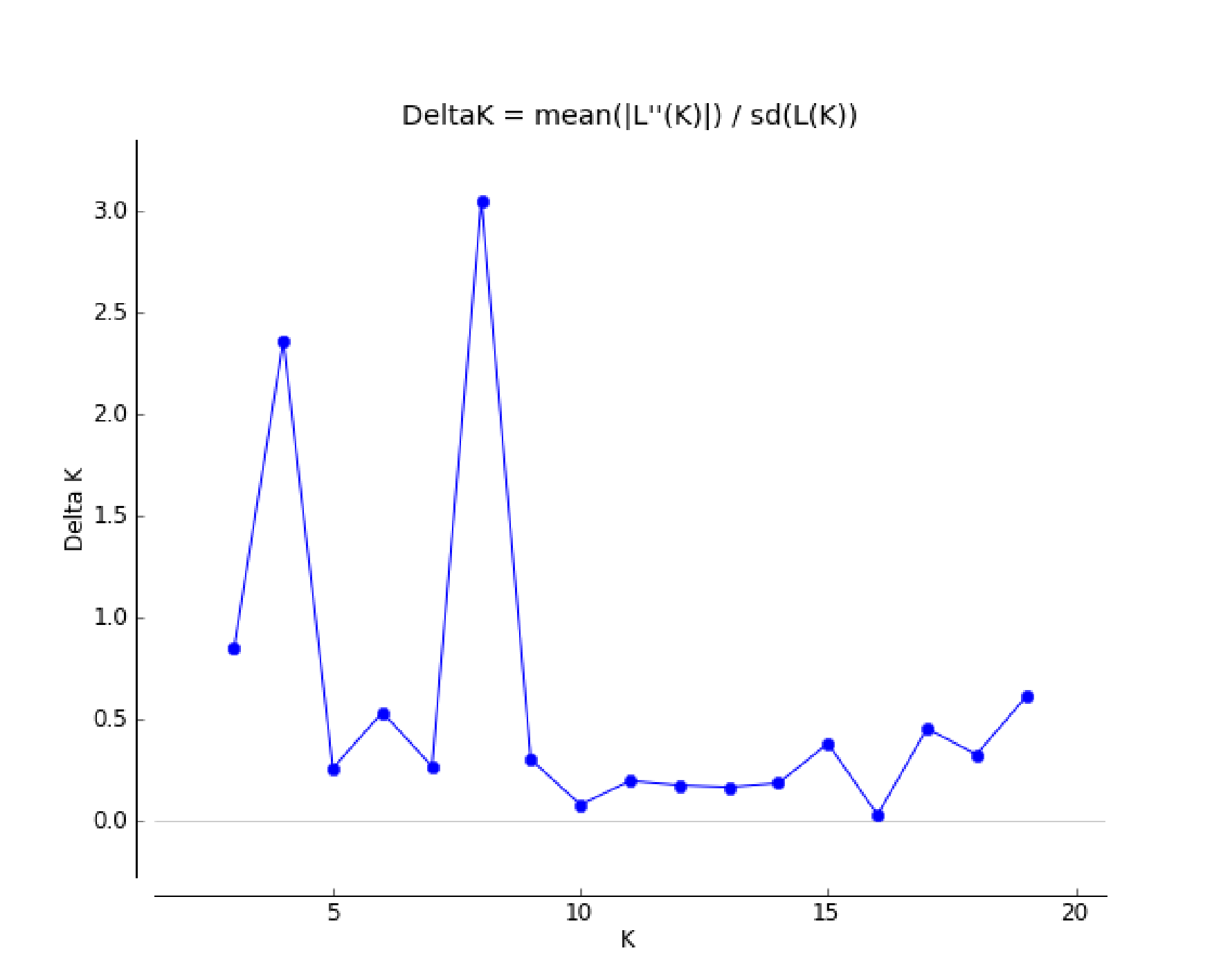

Supplement: Supplemental Information 5 [file peerj-09-11953-s005.png]

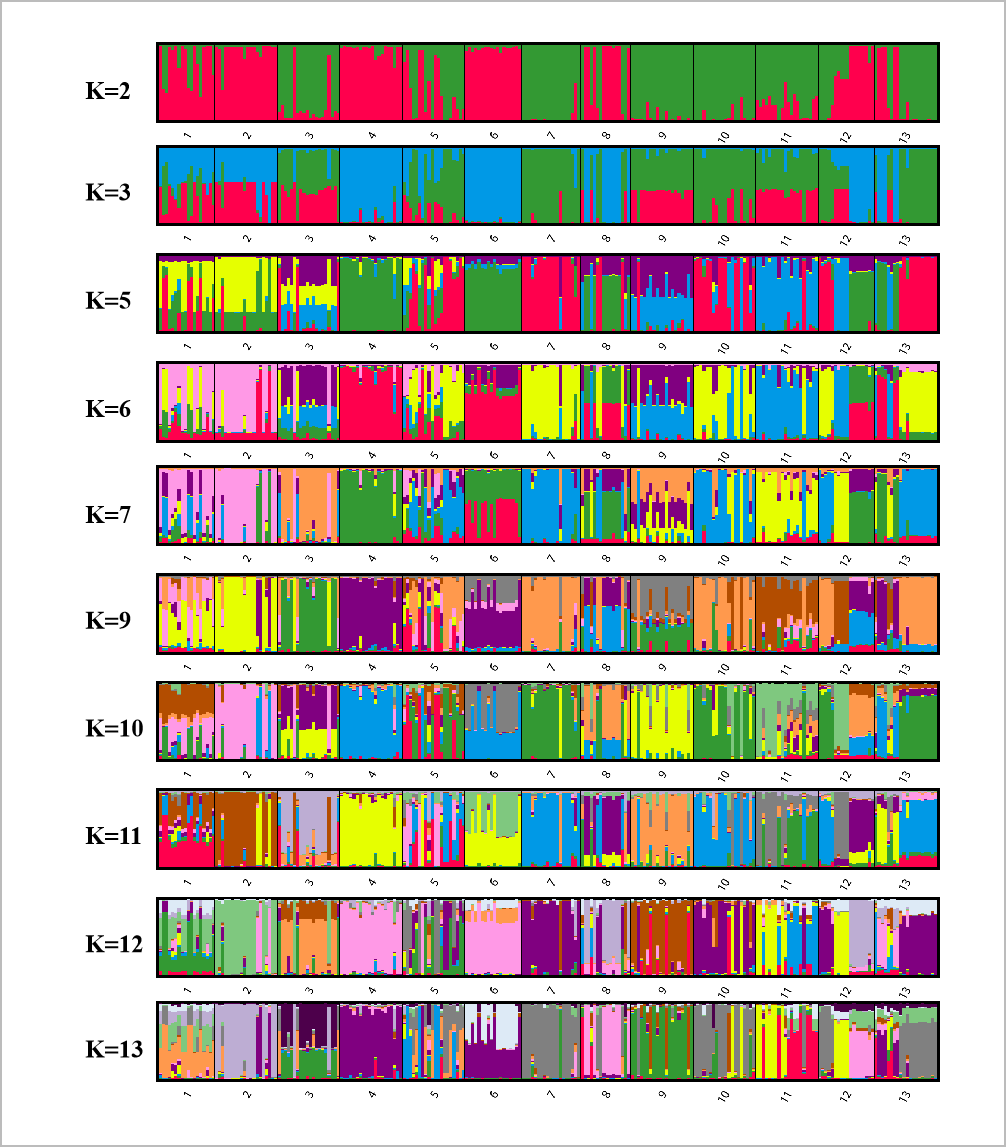

Supplement: Supplemental Information 6 [file peerj-09-11953-s006.png]
