# Supplementary material for: Non-linear genetic diversity and notable population differentiation caused by low gene flow of bermudagrass [Cynodon dactylon (L.) Pers.] along longitude gradients
Source: PeerJ. 2021 Aug 17;9:e11953. doi: 10.7717/peerj.11953 (PMC8378333; doi:10.7717/peerj.11953)
Supplement: Supplemental Information 7 [file peerj-09-11953-s007.docx]

Table S1. Genetic diversity of bermudagrass with different ploidy levels

| Ploidy level | N | He | I | PIC |
| --- | --- | --- | --- | --- |
| 2 | 11 | 0.2478 | 0.3745 | 72.38% |
| 3 | 13 | 0.2105 | 0.3181 | 61.9% |
| 4 | 104 | 0.2453 | 0.3829 | 92.38% |
| 5 | 45 | 0.2691 | 0.4102 | 86.67% |
| 6 | 41 | 0.2598 | 0.3986 | 86.67% |
| Total | 214 | 0.263 | 0.4077 | 100.00% |

He, Nei's gene diversity index; I, Shannon's diversity index; PIC, Polymorphic information content.
